# The *SEPS1* G-105A Polymorphism Is Associated with Risk of Spontaneous Preterm Birth in a Chinese Population

**DOI:** 10.1371/journal.pone.0065657

**Published:** 2013-06-11

**Authors:** Yan Wang, Xiao Yang, Yong Zheng, Zhi-Hao Wu, Xiao-Ai Zhang, Qiu-Ping Li, Xi-Yu He, Chun-Zhi Wang, Zhi-Chun Feng

**Affiliations:** 1 BaYi Children’s Hospital, General Military Hospital of Beijing PLA, P. R. China; 2 The 309 Hospital of PLA, Beijing, P. R. China; 3 Department of Infectious Disease Control, Beijing Institute of Disease Control and Prevention, Beijing, P. R. China; 4 State Key Laboratory of Pathogen and Biosecurity, Beijing Institute of Microbiology and Epidemiology, Beijing, P. R. China; University of Milan, Italy

## Abstract

Inflammation plays an important role in the etiology and pathophysiology of spontaneous preterm birth (SPTB), and selenoprotein S (SEPS1) is involved in regulating the inflammatory response. Recently the G-105A promoter polymorphism in *SEPS1* was shown to increase pro-inflammatory cytokine expression. We examined whether this functional polymorphism was related to the risk of SPTB in a Chinese population. We also examined the impact of premature rupture of membranes (PROM) on susceptibility to SPTB. The *SEPS1* G-105A polymorphism was genotyped in 569 preterm singleton neonates and 673 term neonates by polymerase chain reaction-restriction fragment length polymorphism (PCR-RFLP) analysis. *χ* ^2^ tests and logistic regression analyses were used to calculate the odds ratios (ORs) and 95% confidence intervals (95% CIs). We observed that, compared with the GG genotype, –105A positive genotypes (GA + AA genotypes) were associated with significantly increased susceptibility to SPTB (adjusted OR, 1.87; 95% CI, 1.36–2.57; *P*<0.001). The –105A positive genotypes were also significantly associated with increased susceptibility to SPTB, both in the patients with PROM (adjusted OR, 2.65; 95% CI, 1.73–4.03; *P*<0.001) and in those without PROM (adjusted OR, 1.56; 95% CI, 1.09–2.24; *P* = 0.015). The –105A positive genotypes were also significantly associated with increased susceptibility to SPTB between extremely preterm neonates and controls (adjusted OR, 4.46; 95% CI, 1.86–10.73; *P* = 0.002) and between moderately preterm neonates and controls (adjusted OR, 1.76; 95% CI, 1.25–2.47; *P* = 0.001). Our findings suggest that the *SEPS1* G-105A polymorphism contributes to the risk of developing SPTB in a Chinese population.

## Introduction

Preterm birth (PTB), defined as birth before 37 complete weeks of gestation, is the major cause of neonatal mortality and morbidity. PTB is associated with over 75% of long-term morbidity, including cerebral palsy, developmental delay, retinopathy of prematurity, and hearing and vision problems. PTB is a global problem, with approximately 15 million premature births occurring annually across the globe. This number is rising according to the World Health Organization (http://www.who.int/mediacentre/factsheets/fs363/en/). China contributes 7.8% of these premature births, with nearly 1.1 million preterm infants being born there every year, second only to India [1]. PTB is an important problem in obstetrics, and has become a major public health concern in China.

Genetic factors are known contributors to spontaneous preterm birth (SPTB) [2]. Epidemiological studies suggest that SPTB clusters in families [3]. Moreover, there are significant racial/ethnic disparities in the incidence of SPTB, with African Americans running more than twice as great a risk of SPTB as European-American women. In addition, association studies have identified a number of genetic polymorphisms related to infection, inflammation and innate immune systems that are risk factors for SPTB [4], [5], [6], [7], [8], [9]. Despite these advances, the alleles accounting for the bulk of genetic susceptibility to SPTB remain undiscovered, particularly in Chinese populations. Identifying susceptibility genes conferring increased risks for SPTB would advance the development of solutions to preventing SPTB and would help to clarify the causes and mechanisms of this disease.

Evidence exists that inflammation plays a role in the pathophysiology of SPTB [10], [11], [12]. Given that inflammation contributes to the initiation of SPTB, genes encoding proteins involved in the regulation of inflammatory mediators are plausible candidate genes. Selenoprotein S (SEPS1, gene aliases: SELS, VIMP, TANIS) is a novel selenoprotein, and it impacts the immune and inflammatory signal pathways [13]. SEPS1 has been classified as a new endoplasmic reticulum (ER) membrane protein that moves misfolded proteins from the ER to the cytosol and prevents stress responses that activate the inflammatory cascade [14]. Thus, it is expected that genetic polymorphisms affecting SEPS1 gene transcription and subsequent SEPS1 expression levels might contribute to the development and progression of inflammatory disorders.

A previous study discovered that a functional single nucleotide polymorphism (SNP) in the *SEPS1* promoter region (G-105A, rs28665122) impairs SEPS1 expression and amplifies the production of inflammatory cytokines such as tumor necrosis factor-α (TNF-α), interleukin-6 (IL-6), and interleukin-1β (IL-1β) [13]. Recently, studies have shown that the G-105A polymorphism is associated with risks across a wide spectrum of diseases [15], [16], [17], [18], [19], [20]. The role of the *SEPS1* G-105A polymorphism in SPTB, however, has never been specifically investigated. In the present study we examined whether a functional *SEPS1* G-105A promoter polymorphism has any bearing on SPTB risk in a Chinese population.

## Methods

### Study Population

Subjects in this study were offspring of women receiving obstetrical care at the BaYi Children’s Hospital at General Hospital of Beijing PLA. Subjects were recruited sequentially between Jan. 2009 and May 2011. All subjects were unrelated ethnic Han Chinese residing in Beijing and its surrounding regions. Cases were neonates from pregnancies complicated by SPTB. Based on gestational age, SPTB is dividing into three sub-categories: moderately preterm (33 to 36 completed weeks of gestation), very preterm (<32 weeks) and extremely preterm (<28 weeks). Controls were randomly selected from singleton pregnancies delivered at term from mothers with no prior history of premature rupture of membranes (PROM) or PTB. The diagnosis of PROM was based on pooling of amniotic fluid in the vagina, amniotic fluid ferning patterns, and a positive nitrazine test. Pregnancies with fetal anomalies, trauma, connective tissue diseases, preeclampsia, intrauterine growth restriction, fetal distress, antepartum hemorrhage, or medical complications requiring induction of labor were excluded from both the case and control groups. Pregnancies who treated with antenatal steriods, tocolytics, progesterone, antibiotics, or undergone cerclage upon presentation of preterm were also excluded from both the case and control groups.

For each participant, a neonatal peripheral blood sample was collected into tubes containing EDTA immediately after delivery. We stored the whole blood samples at 4°C upon collection. Then, all whole blood samples were stored at −80°C until genomic DNA extraction. At recruitment, demographic factors and medical history were collected using a structured questionnaire. This study was performed with the approval of the Ethical Committee of General Hospital of Beijing PLA and was conducted according to the principles expressed in the Declaration of Helsinki. At recruitment, written informed consent was obtained from all participants’ guardians.

### *SEPS1* G-105A Genotyping

Genomic DNA was extracted from whole blood specimens using the RelaxGene Blood DNA System (TianGen Biotech Co. Ltd.) according to the manufacturer’s instructions. DNA samples were diluted to 10 ng/µL and distributed to 96-well plates (each 96-well plate contains 94 samples and 2 no-DNA controls). We then analyzed samples for the *SEPS1* G-105A genotype using polymerase chain reaction-restriction fragment length polymorphism (PCR-RFLP). Briefly, the PCR assay was performed in GeneAmp PCR System 9700 (Applied Biosystems). The primers 5′-TCCTTGGCTTCAGTGTCCAAT-3′ and 5′-CGCGGACAGAGACTCCTCTT-3′ were used to amplify the target region containing the G-105A polymorphism. PCR was performed with a 25 µL reaction mixture containing approximately 20 ng DNA, 0.2 µM of each primer, 0.2 mM deoxynucleoside triphosphates, and 0.5 unit Ex Taq Polymerase in 1X reaction buffer (Takara BioTech, Dalian, China). The amplification reaction was carried out in the following conditions: an initial melting step of 2 min at 95°C, followed by 35 cycles of 30 sec at 94°C, 30 sec at 57°C and 30 sec at 72°C with a final elongation of 7 min at 72°C. The reaction yielded a 370 bp amplicon. An aliquot (5 µL) of PCR product was digested with 4 unit of 4 *Msc*I (New England Biolabs) and separated on a 2.5% agarose gel. The presence of the *A* allele creates an *Msc*I restriction site; digested amplicons from AA homozygotes appear as a 233 bp and a 137 bp band, homozygotes for the *G* allele appear as a 370 bp band, and heterozygotes have all three of these bands. To ensure quality control, genotyping was performed blinded to the case/control status of the subjects; 15% masked, random samples from cases (n = 85) and controls (n = 101) were validated by direct sequencing, and the results were 100% concordant.

### Statistical Analysis

Genotype and allele frequencies for the G-105A polymorphism were determined by gene counting. The fitness to Hardy-Weinberg equilibrium was tested using the random-permutation procedure implemented in the Arlequin package (http://lgb.unige.ch/arlequin). The homogeneity of baseline characteristics between the two groups was tested by the *χ* ^2^ test or Fisher exact test for categorical variables, as well as by the Mann-Whitney U test for continuous variables. The single allelic and single genotype frequencies in each of the two groups were analyzed using *χ* ^2^ test. Association between the G-105A polymorphism and risk of SPTB were estimated by use of *χ* ^2^ test and logistic regression analyses. Odds ratios (ORs) and 95% confidence intervals (CIs) were used to measure the strength of association. An association was considered significant at a *P* value of <0.05, and all statistical tests were two-sided. These analyses were performed using SPSS software (version 15.0; SPSS Inc.). The meta-analyses were performed using Review Manager version 5.0 (The Nordic Cochrane Centre, The Cochrane Collaboration, Copenhagen, Denmark). Statistical heterogeneity among studies was evaluated using the *χ* ^2^ test, *P* values, and *I* ^2^ statistics [21].

## Results

### Demographic Characteristics

All subjects were of Chinese Han descent. The baseline characteristics of the study population, including 569 infants (SPTB <37 weeks) and 673 controls, are shown in Table 1. There were no statistically significant differences in maternal age, gravidity, parity or neonatal gender between the SPTB and control cohorts. Significant differences between cases and controls were observed for gestational age at delivery (week) (*P*<0.001), birth weight (g) (*P*<0.001), APGAR 1 (1 min after birth) (*P*<0.001), and APGAR 5 (5 min after birth) (*P*<0.001).

**Table 1:** Distributions of select characteristics among preterm neonates and controls.

Variable^a^	Cases (n = 569)	Controls (n = 673)	*P* value^b^
Maternal age at delivery (year)	28 (26–30.5)	29 (26–31)	0.350
Gravidity	1 (1–6)	1 (1–7)	0.779
Parity	1 (1–3)	1 (1–3)	0.074
Gestational age at delivery (week)	34.0 (32.0–35.4)	39.0 (38.2–39.6)	<0.001
Neonatal birth weight (g)	2060 (1700–2500)	3400 (3045–3650)	<0.001
Neonatal sex			0.595
Boy	318 (55.9)	366 (54.4)	
Girl	251 (44.1)	307 (45.6)	
1 min Apgar score	10 (9–10)	10 (10–10)	<0.001
5 min Apgar score	10 (9–10)	10 (10–10)	<0.001
Sub-categories of SPTB			
extremely preterm (<28 weeks)	26 (4.6)		
very preterm (28 to <32 weeks)	97 (17.0)		
moderate to late preterm (32 to <37 weeks)	446 (78.4)		
PROM	171 (30.1)		

Abbreviations: PROM, premature rupture of membranes.

a Results are expressed as the median (25th–75th percentile) or as absolute numbers of patients (percentage).

b Fisher exact test or *χ^2^* test for categorical variables and the Mann-Whitney U test for continuous variables.

### Analysis of *SEPS1* G-105A Polymorphism in a Chinese Han Population

Genotyping results are presented in Table 2. The observed genotype frequencies of the *SEPS1* G-105A polymorphism were in respective Hardy-Weinberg equilibrium for both cases and controls (all *P*>0.05, data not shown). In the overall sample, the A allele was more prevalent among case infants (10%) than among control subjects (6%) (*χ* ^2^ = 18.15, *P*<0.001, *df* = 1). The frequencies of the GG, GA and AA genotypes among SPTB neonates varied significantly from those among controls (*χ* ^2^ = 18.92, *P*<0.001, *df* = 2). We used a dominant genetic model (frequency of GA heterozygotes plus frequency of AA homozygotes vs. frequency of GG homozygotes). Subjects carrying the –05A allele (GA + AA genotypes) had an elevated risk of SPTB compared to those with the GG genotype (OR, 1.85; 95% CI, 1.35–2.55; *P*<0.001). In multivariate logistic regression analysis with maternal age at delivery (year) and neonatal sex adjusted, significant association was also observed between SPTB and G-105A polymorphism (adjusted OR, 1.87; 95% CI, 1.36–2.57; *P*<0.001).

**Table 2:** Genotype and allele frequencies of the *SEPS1* G-105A polymorphism in preterm neonates and controls.

	Cases (%)	Controls (%)	Crude		Adjusted	
	(n = 569)	(n = 673)	OR (95% CI)	*P* value	OR (95% CI)	*P* value
Genotypes						
GG	458 (80.9)	597 (88.7)	Reference		Reference	
GA	99 (17.5)	75 (11.1)				
AA	9 (1.6)	1 (0.2)				
GA + AA	108 (19.1)	76 (11.3)	1.85 (1.35–2.55)^a^	<0.001^a^	1.87 (1.36–2.57)^c^	<0.001^c^
Alleles						
* G* allele	0.90	0.94		<0.001^b^		
* A* allele	0.10	0.06				

NOTE: Due to genotyping failure, the actual sample size, respectively, was 566 and 673 for cases and controls.

Abbreviations: OR, odds ratio; CI, confidence interval.

a ORs and *P* values obtained against reference by *χ* ^2^ test (2×2).

b Two-sided *χ^2^* test for distribution of allelic frequencies (*df* = 1).

c ORs and *P* values were calculated by multivariate logistic regression, adjusted for maternal age at delivery (year) and neonatal sex.

The associations between the G-105A polymorphism and susceptibility to SPTB were further examined by stratifying patients by PROM status (Table 3). The –105A positive genotypes were significantly associated with increased susceptibility to SPTB both in the patients with PROM (OR, 2.64; 95% CI, 1.73–4.02; *P*<0.001) and in those without PROM (OR, 1.55; 95% CI, 1.08–2.21; *P* = 0.017). In multivariate logistic regression analysis with maternal age at delivery (year) and neonatal sex adjusted, significant association was observed between susceptibility to SPTB and –105A positive genotypes both in the patients with PROM (adjusted OR, 2.65; 95% CI, 1.73–4.03; *P*<0.001) and in those without PROM (adjusted OR, 1.56; 95% CI, 1.09–2.24; *P* = 0.015).

**Table 3:** Genotype and allele frequencies of the *SEPS1* G-105A polymorphism in preterm neonates delivered with or without PROM and controls.

	Cases (%)	Controls (%)	Crude		Adjusted	
			OR (95% CI)	*P* value	OR (95% CI)	*P* value
With PROM	(n = 171)	(n = 673)				
Genotypes						
GG	128 (74.8)	597 (88.7)	Reference		Reference	
GA	38 (22.2)	75 (11.1)				
AA	5 (2.9)	1 (0.2)				
GA + AA	43 (25.1)	76 (11.3)	2.64 (1.73–4.02)^a^	<0.001^a^	2.65 (1.73–4.03)^c^	<0.001^c^
Alleles						
* G* allele	0.86	0.94		<0.001^b^		
* A* allele	0.14	0.06				
Without PROM	(n = 398)	(n = 673)				
Genotypes						
GG	330 (83.5)	597 (88.7)	Reference		Reference	
GA	61 (15.4)	75 (11.1)				
AA	4 (1)	1 (0.2)				
GA + AA	65 (16.5)	76 (11.3)	1.55 (1.08–2.21)^a^	0.017^a^	1.56 (1.09–2.24)^c^	0.015^c^
Alleles						
* G* allele	0.91	0.94		0.008^b^		
* A* allele	0.09	0.06				

NOTE: Due to genotyping failure, the actual sample size, respectively, was 566 and 673 for cases and controls.

Abbreviations: OR, odds ratio; CI, confidence interval.

a ORs and *P* values obtained against reference by *χ* ^2^ test (2×2).

b Two-sided *χ^2^* test for distribution of allelic frequencies (*df* = 1).

c ORs and *P* values were calculated by multivariate logistic regression, adjusted for maternal age at delivery (year) and neonatal sex.

The frequencies of G-105A polymorphism genotypes were also compared between 425 preterm neonates with low birthweight and 144 neonates with normal birthweight (Table 4). There were no significant differences in the distribution of genotypes and alleles within the two groups.

**Table 4:** Genotype and allele frequencies of the *SEPS1* G-105A polymorphism in preterm neonates delivered with or without low birthweight.

	Birthweight <2500 g (n = 425)	Birthweight ≧2500 g (n = 144)	Crude		Adjusted	
			OR (95% CI)	*P* value	OR (95% CI)	*P* value
Genotypes						
GG	343 (81.1)	115 (80.4)	Reference		Reference	
GA	74 (17.5)	25 (17.5)				
AA	6 (1.4)	3 (2.1)				
GA + AA	80 (18.9)	28 (19.6)	0.96 (0.59–1.55)^a^	0.861^a^	0.96 (0.59–1.55)^c^	0.861^c^
Alleles						
* G* allele	0.90	0.89		0.746^b^		
* A* allele	0.10	0.11				

NOTE: Due to genotyping failure, the actual sample size was 566 for cases.

Abbreviations: OR, odds ratio; CI, confidence interval.

a ORs and *P* values obtained against reference by *χ* ^2^ test (2×2).

b Two-sided *χ^2^* test for distribution of allelic frequencies (*df* = 1).

c ORs and *P* values were calculated by multivariate logistic regression, adjusted for maternal age at delivery (year) and neonatal sex.

We also analyzed the association between the controls and three gestational age groupings: extremely preterm neonates, very preterm neonates, and moderately preterm neonates (Table 5). The –105A positive genotypes were significantly associated with increased susceptibility to SPTB between extremely preterm neonates and controls (adjusted OR, 4.46; 95% CI, 1.86–10.73; *P* = 0.002). There was a significant association between moderately preterm neonates and controls as well (adjusted OR, 1.76; 95% CI, 1.25–2.47; *P* = 0.001). However, only a borderline significant association was observed between very preterm neonates and controls (adjusted OR, 1.81; 95% CI, 1.03–3.19; *P* = 0.050).

**Table 5:** Genotype and allele frequencies of the *SEPS1* G-105A polymorphism in controls and three sub-categories of SPTB neonates.

	Cases (%)			Controls (%) (n = 673)	OR (95% CI)			*P* value		
	P1 (n = 26)	P2 (n = 97)	P3 (n = 446)		P1 vs. Controls	P2 vs. Controls	P3 vs. Controls	P1 vs. Controls	P2 vs. Controls	P3 vs. Controls
Genotypes										
GG	16 (64.0)	79 (81.4)	363 (81.8)	597 (88.7)	Reference	Reference	Reference	Reference	Reference	Reference
GA	9 (36.0)	18 (18.6)	72 (16.2)	75 (11.1)						
AA	0	0	9 (2.0)	1 (0.2)						
GA + AA	9 (36.0)	18 (18.6)	81 (18.2)	76 (11.3)						
GG vs. GA + AA					4.42 (1.89–10.35)^a^	1.79 (1.02–3.15)^a^	1.75 (1.25–2.46)^a^	0.002^a^	0.053^a^	0.001^a^
GG vs. GA + AA					4.46 (1.86–10.73)^b^	1.81 (1.03–3.19)^b^	1.76 (1.25–2.47) b	0.002^b^	0.050^b^	0.001^b^
Alleles										
* G* allele	0.82	0.91	0.90	0.94				<0.001^c^	0.054^c^	<0.001^c^
* A* allele	0.18	0.09	0.10	0.06						

NOTE: Due to genotyping failure, the actual sample size, respectively, was 566 and 673 for cases and controls. P1, extremely preterm neonates; P2, very preterm neonates; P3, moderately preterm neonates.

Abbreviations: OR, odds ratio; CI, confidence interval.

a ORs and *P* values obtained against reference by *χ* ^2^ test (2×2).

b ORs and *P* values were calculated by multivariate logistic regression, adjusted for maternal age at delivery (year) and neonatal sex.

c Two-sided *χ^2^* test for distribution of allelic frequencies (*df* = 1).

**Figure 1:**
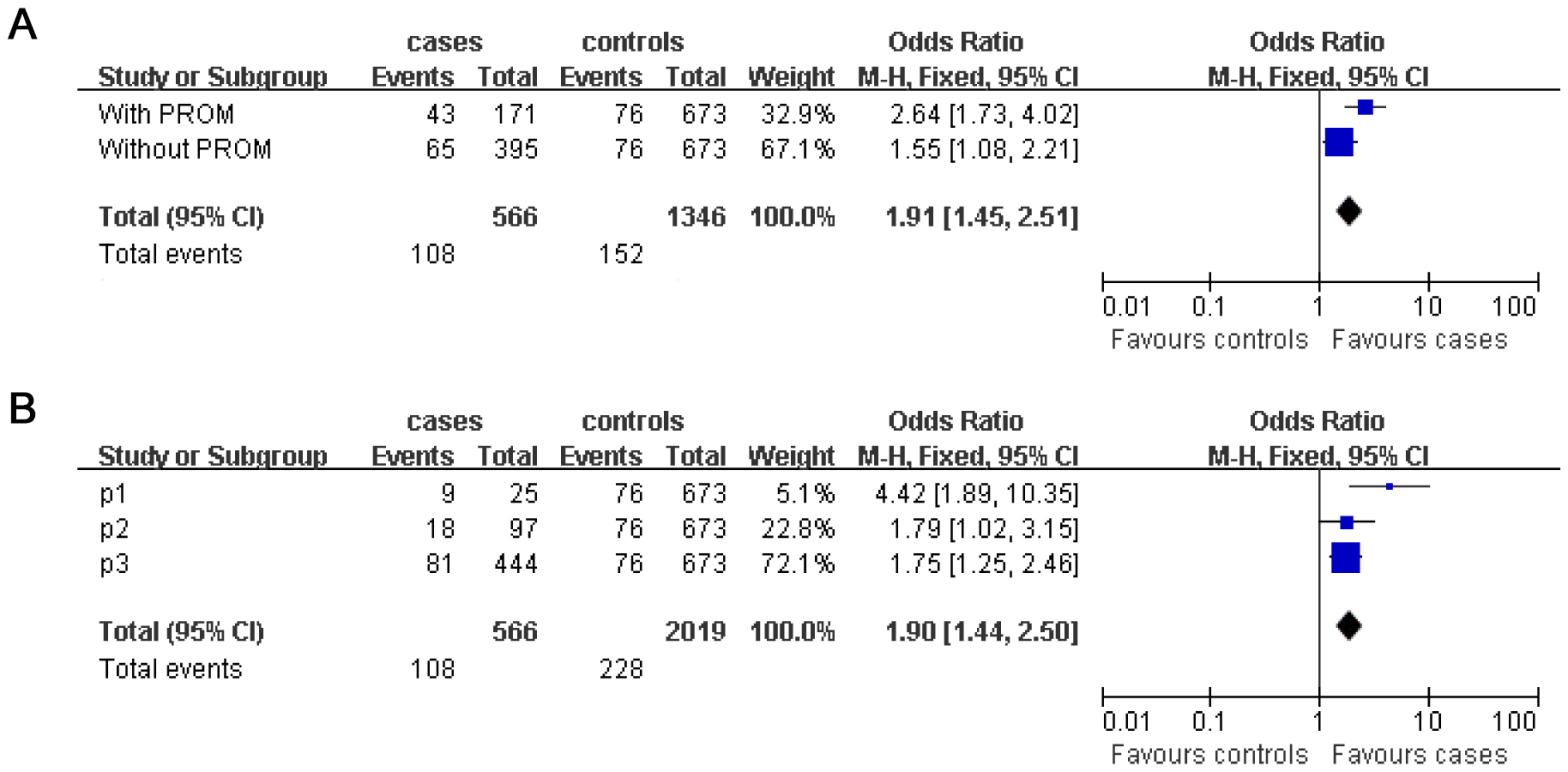
Forest plot of meta-analysis on the association between the *SEPS1* G-105A polymorphism and SPTB risk. We plot the odds ratio (OR) (blue square) and the 95% CI (horizontal blue line) for different group of patients compared with control respectively. A vertical dashed dark line indicates the final OR across all groups. The top bars represent data from each group and the blue diamond below them summarizes their meta-analyzed effect. The area of each square is proportional to the weight of each study in the meta-analysis. (*A*). Forest plot of meta-analysis on the association between the *SEPS1* G-105A polymorphism and SPTB risk in preterm neonates delivered with or without PROM and controls. Overall, the meta-analysis gave a overall OR of 1.91, 95% CI = 1.45–2.51. (*B*) Forest plot of meta-analysis on the association between the *SEPS1* G-105A polymorphism and SPTB risk in controls and three sub-categories of SPTB neonates. Overall, the meta-analysis gave a overall OR of 1.90, 95% CI = 1.90–2.50. P1, extremely preterm neonates; P2, very preterm neonates; P3, moderately preterm neonates.

To examine whether there was a possible difference in the effect of *SEPS1* G-105A polymorphism on SPTB between stratified analysis of PROM, three gestational age groupings and overall analysis, we performed a meta-analysis. For comparison between subjects carrying the GG genotype and subjects carrying the −105A positive genotypes, the meta-analysis of data in preterm neonates delivered with or without PROM and controls showed significant association between *SEPS1* G-105A polymorphism and SPTB (OR = 1.91, 95% CI, 1.45–2.51, Figure 1A). Meta-analysis of data in three gestational age groupings and controls also revealed a significant association (OR = 1.90, 95% CI, 1.44–2.50, Figure 1B). Thus, the meta-analysis further indicates that the –105A positive genotypes were significantly associated with increased susceptibility to SPTB.

## Discussion

In the present study, we attempted to examine whether the *SEPS1* G-105A polymorphism was related to an increased risk of SPTB. We performed a case-control study to test the association between SPTB and *SEPS1* G-105A polymorphism in the Han of North China. We detected strong evidence of an association between the *SEPS1* G-105A polymorphism and SPTB. To the best of our knowledge, this is the first report of a genetic association between the *SEPS1* polymorphism and susceptibility to SPTB, confirming the initial hypothesis that SEPS1 may play a role in the pathogenesis of this disorder.

The genetic association between the *SEPS1* G-105A polymorphism and occurrence of SPTB is biologically plausible. It is well established that inflammation is associated with SPTB [10], [11], [12]. During recent years, the link between polymorphisms in genes encoding cytokines involved in inflammatory mechanisms has received increased attention from researchers [4], [5], [6], [7]. However, no study has yet explored association between SPTB and SEPS1, which is an upstream regulatory factor of inflammatory cytokine [14]. In the study by Curran, et. al., the *SEPS1* G-105A polymorphism was strongly associated with circulating levels of pro-inflammatory cytokines such as IL-1β, IL-6 and TNF-α, as well as with *SEPS1* gene expression levels in humans [13]. The −105A allele reduced promoter activity in the *SEPS1* gene and was significantly associated with increased cellular cytokine production and release. In our study population, the carriers of the *SEPS1*−105A allele were overrepresented in cases relative to controls, suggesting that the −105A allele is an at-risk allele for SPTB. This hypothesis was in line with the function of the *SEPS1* G-105A polymorphism mentioned above. Hence, given the role of *SEPS1* in inflammation, one might expect individuals who carry the −105A allele, and thus exhibit decreased expression of *SEPS1*, to have a higher susceptibility to developing SPTB.

PROM is characterized by membrane rupture prior to the onset of full-term labor, complicating 1–4% of all pregnancies. In our study the association of SPTB with risk was stronger for patients with PROM than for patients without PROM. Activation of SPTB is thought to occur via multiple pathways and mechanisms, though PROM is the leading identifiable cause of PTB [22]. Moreover, inflammation has been implicated in the etiologies of both SPTB and PROM [22], [23]. The association of SPTB and PROM with inflammation and elevated body fluid concentrations of inflammatory cytokines has been frequently observed [24], [25], [26]. Because common pathways have been observed in SPTB, PROM and altered molecular routes of inflammation, it is biologically rational that the association with the risk of SPTB is stronger for patients with PROM than for patients without PROM.

Considering that SPTB etiologies vary based on gestational age at delivery, we analyzed the associations between controls and three gestational age groupings: extremely preterm (<28 weeks), very preterm (<32 weeks), and moderately preterm (33 to 36 completed weeks of gestation). We found that the G-105A polymorphism was significantly associated with SPTB in the extremely preterm and moderately preterm groups, while we observed only a borderline significant association in the very preterm group. Interestingly, several other studies also suggested differences of susceptibility between gestational age groupings [26], [27], [28]. Thus, it is plausible to assume that the associations between *SEPS1* G-105A polymorphism and SPTB are different based on the gestational age at delivery. However, considering the limited sample size of extremely preterm neonates (n = 26) in our study, this assumption warrants further confirmation in future studies.

In reviewing the results of this study, two potential limitations should be kept in mind. Firstly, in our study, the influence of maternal genomes on predispositions to SPTB was not considered. SPTB is a function of both maternal and fetal risk factors and their interactions at the genetic and biomarker levels. Additional well-designed case-control studies that include both maternal and fetal genomes are warranted to more fully understand the roles of the *SEPS1* polymorphism in the etiology of SPTB. Secondly, we cannot rule out that the presence of polymorphisms in other genes, especially other genes involved in the inflammation, could affect the occurrence of the SPTB. We selected *SEPS1* G-105A polymorphism because it was strongly associated with circulating levels of pro-inflammatory cytokines. However, the regulation of inflammation is complex, with other genes warranting investigation. Recently, other investigators have used proteomics [29] and microarray [30], [31] to identify markers for SPTB in a more comprehensive manner. Thus, further studies that cover more genes involved in the inflammation are warranted to fully clarify the etiology of SPTB.

In conclusion, we found that the *SEPS1* G-105A polymorphism was associated with the risk of SPTB in a Chinese population. If confirmed by other studies, our findings of genetic factors contributing to the pathogenesis of SPTB may have implications for the screening and treatment of this disorder.
